# Unusual Case of a Talon Cusp on a Supernumerary Tooth in Association with a Mesiodens

**DOI:** 10.5681/joddd.2010.016

**Published:** 2010-06-24

**Authors:** Prashant Babaji, Firoza Sanadi, Mahesh Melkundi

**Affiliations:** ^1^ Department of Pedodontics, SPIDM Dental College, Lucknow, Utter Pradesh, India; ^2^ Professor & Head Department of Pedodontics & Preventive Dentistry, Sardar Patel Post Graduate Institute of Dental and Medical Sciences, Lucknow; ^3^ Lecturer Department of Oral pathology College of Dental Sciences Rahu, Indure Madya Pradesh, India

**Keywords:** Talon cusp, supernumerary tooth, mesiodens

## Abstract

Talon cusp is an accessory cusp similar to a projection, extending from the cingulum or cemento-enamel junction to the incisal edge. It occurs on labial or palatal surfaces of primary or permanent anterior teeth in both arches. This accessory cusp can occur as an isolated entity or in association with other dental anomalies. Occurrence of a talon cusp on supernu-merary teeth is rare and uncommon. This paper reports an unusual case of a talon cusp on a supernumerary tooth in association with mesiodens.

## Introduction

Talon cups are morphologically well-delineated, accessory talon-shaped cusps, projecting from the lingual or facial surface of the crown of incisors and extending at least half the distance from the cementoenamel junction to the incisal edge.^1^ Talon cusp can occur in maxillary or mandibular anterior teeth in both the primary and permanent dentition. This accessory cusp can occur as an isolated entity or in association with other dental anomalies. Occurrence of talon cusp on supernumerary teeth is extremely rare. Supernumerary teeth may vary in shape, size and structure or they can resemble the adjacent tooth. This accessory cusp shows increased predilection for males and the maxilla.^2,3^ Reported review of literature has shown that talon cusp exhibits a prevalence of 75% in the permanent dentition compared to 25% in the primary dentition.^3^ The prevalence of talon cusp varies considerably between different ethnic groups. It has a prevalence rate of 0.06% in Mexican, 7.7% in North Indian, 0.17% in American and 2.5% in Hungarian children.^4,5,6,7^

The exact etiology of this condition remains unknown. It is thought to occur during morphodifferentiation stage as a result of outward folding of inner enamel epithelial cells (precursors of ameloblasts) and transient focal hyperplasia of mesenchymal dental papilla (precursors of odontoblasts) or a combination of genetic and environmental factors (multifactorial).^2^

## Case Report

**Figure 1:**
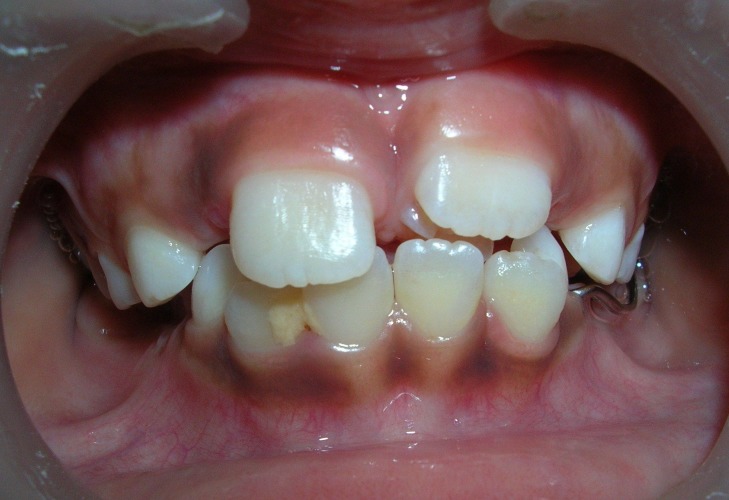
A talon cusp on a maxillary supernumerary tooth resulting in slight labial position of the left central incisor and midline diastema.

**Figure 2:**
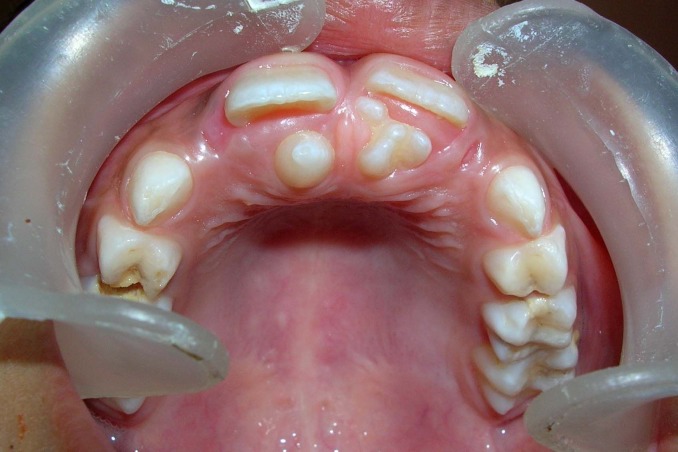
A talon cusp on the palatal surface of maxillary supernumerary tooth on the left side and mesiodens on the right side, palatal to central incisors.

**Figure 3:**
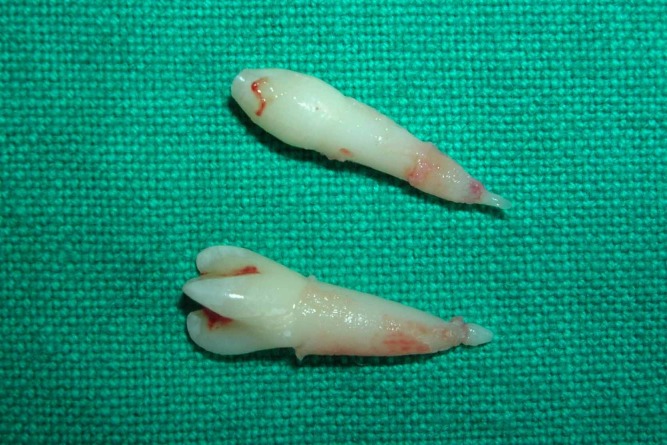
Extracted supernumerary tooth and mesiodens. Talon cusp on the palatal surface of the supernumerary tooth, extending from the cementoenamel junction to the incisal edge.

**Figure 4:**
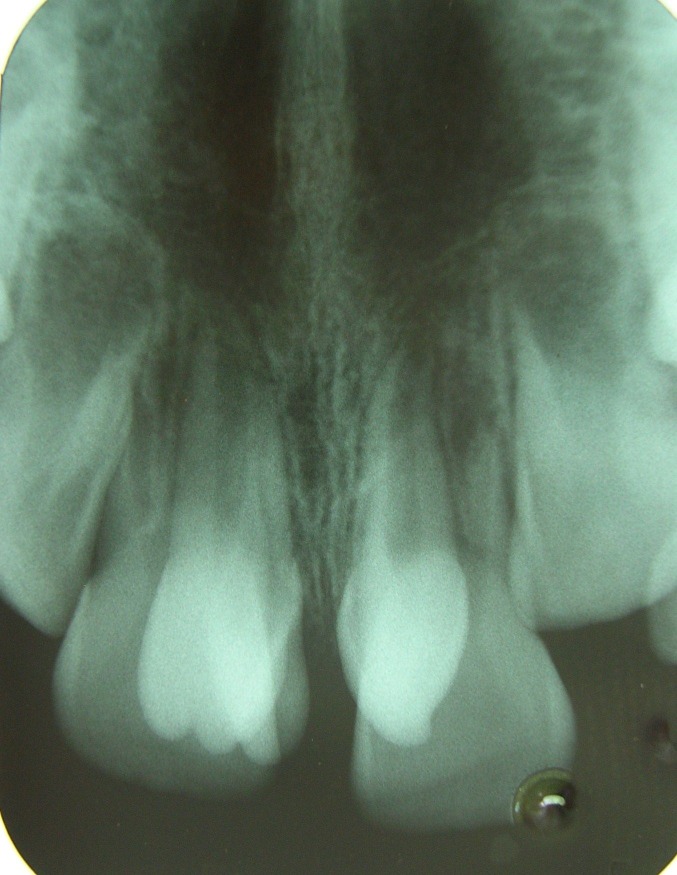
Periapical radiograph shows a V-shaped talon cusp of enamel and dentin with pulp tissue on supernumerary tooth and a mesiodens. The talon cusp extends from the cemento-enamel junction to the incisal edge.

A 6-year-old boy reported to the Department of Pedodontics and Preventive Dentistry, SPPGIDMS, Lucknow, with a complaint of poor esthetics and an additional tooth in the upper jaw. Intraoral examination revealed no soft tissue abnormalities. Maxillary left central incisor was slightly labially positioned with midline diastema because of a palatally positioned supernumerary tooth. A mesiodens was present palatal to maxillary right central incisor (Figure 1 and 2). The supernumerary tooth resembled maxillary central incisor with a mature root and pronounced mamellons and a talon cusp on the palatal surface. The talon cusp was pyramidal in shape and extended from the cementoenamel junction to the incisal edge (Figure 3 and 4).

The periapical radiograph (Figure 4) showed a V-shaped radiopaque structure superimposed on the image of the involved tooth crown, with the tip of the ‘V’ towards the incisal edge. Shallow developmental grooves were present at the junction of the talon cusp with the palatal surface of the affected tooth without any carious lesions. The talon cusp did not interfere with the occlusion because the maxillary and mandibular incisors were not fully erupted. Management included extraction of the taloned supernumerary tooth and mesiodens under local anesthesia.

## Discussion

Talon cusp is an accessory cusp-like structure, which arises during the morphodifferentiation stage of tooth development. This cusp can occur on lingual or labial surfaces of either primary or permanent anterior teeth.^8,9^ Histomorphometric examination has revealed that the talon cusp has normal enamel and dentin with a substantially enlarged pulp tissue, which suggests that talon tooth may be a developmental anomaly originating in the stage of morphodifferentiation.^10^ Using a non-destructive investigation of the taloned crown undercone-beam x-ray computed tomography has revealed pulpal extensionsin two talon cusps.^11^

This anomaly varies widely in shape, size, structure, location and origin. Occurrence of a talon cusp on the labial surface and double labial talon cusps is rare and uncommon.^8^ Occurrence of talon cusps on supernumerary teeth is extremely rare. Supernumerary teeth may vary in shape, size and structure or they can resemble the adjacent tooth. Pubmed and Medline search reveals six cases of talon cusps on supernumerary permanent ^12,13,14^ and primary^11,14,15^ and three cases on supplemental permanent teeth.^16^

Hattab et al^2^ categorized talon cusps into talon, semi-talon and trace talon, according to the extent of the accessory cusp from cementoenamel junction towards the incisal edge. The anomaly appears to be more frequent in Rubinstein-Taybi syndrome,^9^ Mohr syndrome,^17^ Sturge-Weber syndrome^18^ and incontinentia pigmenti.^19^ Talon cusps may be of great clinical importance and early diagnosis may be critical. Small talon cusps are usually asymptomatic and need no treatment. On the other hand, large prominent cusps may cause clinical problems, including poor esthetics, occlusal interference, displacement of the affected tooth, carious lesions in the developmental grooves and pulpal exposure due to cuspal attrition, accidental cuspal fracture, pulpal necrosis, periapical pathoses, periodontal pockets, tongue irritation and possibility of temporomandibular joint pain.,^20-22^

The majority of cases reported in the literature indicate that talon cusp is an isolated anomaly rather than an integral part of any disorder. Talon cusp can be found in isolation or in association with other dental anomalies such as peg-shaped lateral incisors, shovel-shaped incisors, bifid cingulum, unerupted canines and the large cusp of Carabelli, dens invaginatus, supernumeraries and complex odontomas.^2,20,23^ Panoramic radiographs are recommended for exclusion of the association of talon cusps with other abnormalities, including supernumerary teeth, odontomas, and impacted or unerupted teeth.

The present case shows the following features: 1. unusual occurrence of talon cusp on supernumerary teeth; 2. midline diastema; and 3. associated mesiodens. Treatment was planned for extraction of the supernumerary tooth and mesiodens.

## Conclusion

Talon cusp is an odontogenic anomaly arising during the morphodifferentiation stage of tooth development. This accessory cusp can occur on primary or permanent anterior teeth. Occurrence of talon cusp on supernumerary teeth in association with mesiodens is rare and uncommon. Presence of a prominent talon cusp may give rise to various clinical problems, necessitating immediate intervention.
